# Clinical and Laboratory Toxicity after Intra-Arterial Radioembolization with ^90^Y-Microspheres for Unresectable Liver Metastases

**DOI:** 10.1371/journal.pone.0069448

**Published:** 2013-07-24

**Authors:** Maarten L. J. Smits, Andor F. van den Hoven, Charlotte E. N. M. Rosenbaum, Bernard A. Zonnenberg, Marnix G. E. H. Lam, Johannes F. W. Nijsen, Miriam Koopman, Maurice A. A. J. van den Bosch

**Affiliations:** 1 Department of Radiology and Nuclear Medicine, University Medical Center Utrecht, Utrecht, The Netherlands; 2 Department of Medical Oncology, University Medical Center Utrecht, Utrecht, The Netherlands; University of Oklahoma Health Sciences Center, United States of America

## Abstract

**Objective:**

To investigate clinical and laboratory toxicity in patients with unresectable liver metastases, treated with yttrium-90 radioembolization (^90^Y-RE).

**Methods:**

Patients with liver metastases treated with ^90^Y-RE, between February 1^st^ 2009 and March 31^st^ 2012, were included in this study. Clinical toxicity assessment was based on the reporting in patient’s charts. Laboratory investigations at baseline and during a four-month follow-up were used to assess laboratory toxicity according to the Common Terminology Criteria for Adverse Events version 4.02. The occurrence of grade 3–4 laboratory toxicity was stratified according to treatment strategy (whole liver treatment in one session versus sequential sessions). Response assessment was performed at the level of target lesions, whole liver and overall response in accordance with RECIST 1.1 at 3- and 6 months post-treatment. Median time to progression (TTP) and overall survival were calculated by Kaplan-Meier analysis.

**Results:**

A total of 59 patients, with liver metastases from colorectal cancer (n = 30), neuroendocrine tumors (NET) (n = 6) and other primary tumors (n = 23) were included. Clinical toxicity after ^90^Y-RE treatment was confined to grade 1–2 events, predominantly post-embolization symptoms. No grade 3–4 clinical toxicity was observed, whereas laboratory toxicity grade 3–4 was observed in 38% of patients. Whole liver treatment in one session was not associated with increased laboratory toxicity. Three-months disease control rates for target lesions, whole liver and overall response were 35%, 21% and 19% respectively. Median TTP was 6.2 months for target lesions, 3.3 months for the whole liver and 3.0 months for overall response. Median overall survival was 8.9 months.

**Conclusion:**

The risk of severe complications or grade 3–4 clinical toxicity in patients with liver metastases of various primary tumors undergoing ^90^Y-RE is low. In contrast, laboratory toxicity grade 3–4 can be expected to occur in more than one-third of patients without any clinical signs of radiation induced liver disease.

## Introduction

Intra-arterial radioembolization with yttrium-90 microspheres (^90^Y-RE) is an increasingly applied treatment option for patients with unresectable primary or secondary hepatic malignancies, refractory to systemic therapies. The treatment consists of intra-arterial administration of microspheres tagged with or containing yttrium-90 (^90^Y), a radioisotope that emits high-energy beta radiation. In contrast to the normal liver parenchyma, which mainly relies on the portal vein, intrahepatic malignancies mainly depend on the hepatic artery for their blood supply. [1] As a consequence, these tumors can be selectively targeted by instillation of ^90^Y-microspheres in the hepatic artery.

There is growing evidence for an overall beneficial effect of ^90^Y-RE regarding time to progression, overall survival and quality of life in salvage patients with either primary or metastatic hepatic malignancies.[2]–[4] The effect of ^90^Y-RE in terms of tumor response varies widely, with disease control rates (complete response+partial response+stable disease) ranging from 56% –100%. [4] Given the wide variety in tumor response rates, great effort is put into optimal patient selection through the identification of prognostic factors for a favorable outcome after ^90^Y-RE.[5]–[7] Improved selection may increase the efficacy of this therapy and prevent patients from futile treatment and unnecessary toxicity.

Although minimally invasive, ^90^Y-RE is not without adverse effects. Common adverse effects related to ^90^Y-RE are symptoms of the post-embolization syndrome, comprising fatigue, nausea, vomiting, abdominal pain, loss of appetite and fever.[7]–[10] In general, these symptoms appear on the day of treatment and last up to three days after treatment. [11] More serious complications can occur when an excessive radiation dose is applied to non-target tissue. An excessive dose to the healthy liver parenchyma, which can be due to either a high overall administered activity or an unfavorable tumor to non-tumor activity distribution ratio, can cause radiation induced liver disease (RILD). Alternatively, distribution of microspheres in organs other than the liver could cause serious morbidity and even mortality (e.g. radiation pneumonitis or gastric ulceration). These severe complications occur in less than 10% of patients.[12]–[14].

Laboratory toxicity in terms of elevated liver function tests and liver enzymes can be expected after ^90^Y-RE. It is important to monitor laboratory toxicity, because this may be an early indicator for RILD. Relatively little is known, however, about the normal range of laboratory toxicities following ^90^Y-RE in patients who do not develop RILD. The primary objective of this study was to investigate clinical and laboratory toxicity in patients with liver metastases, treated with ^90^Y-RE. Secondary objectives were assessment of tumor response and overall survival.

## Materials and Methods

### Patient Selection

Records of all liver metastases patients who were not participating in a clinical trial and had received a pre-treatment angiographic procedure for treatment with ^90^Y-RE at our institute between February 1^st^ 2009 and March 31^st^ 2012 were retrospectively analyzed. Patients that were eligible for ^90^Y-RE had unresectable liver dominant metastases and had progressive disease under systemic treatment, or were no longer treated systemically due to contraindications. The Medical Ethics Committee of the University Medical Center Utrecht waived the need for informed-consent and approved this study.

### Procedure

^90^Y-RE was carried out over two sessions: a pre-treatment diagnostic angiography and a treatment angiography. Patients were admitted to the hospital on the evening before angiography. They received 1.5 L per 24 h NaCl 0.9% intravenously for pre- and post-hydration. Pre-treatment diagnostic angiography started with selective visceral catheterization (celiac axis and superior mesenteric artery) in order to obtain an angiographic map of the patients’ vascular anatomy. Specific extrahepatic vessels were coil-embolized to prevent ^90^Y-microspheres that were injected into one of the hepatic arteries, to be distributed to visceral organs other than the liver. Arteries that were actively searched for and embolized using coils included the gastroduodenal artery, the right gastric artery, and pancreaticoduodenal vessels and any other relevant arteries depending on the patient’s specific anatomy. Subsequently, 150 MBq technetium-99m-labelled macro-albumin aggregates (^99m^Tc-MAA) were injected into the hepatic artery to simulate the ^90^Y-microspheres distribution. Next, single photon emission computed tomography (SPECT) and planar nuclear imaging were performed. In order to assess whether part of the dose was deposited in abdominal organs other than the liver, the SPECT images were analyzed after fusion with computed tomography (CT). Planar nuclear imaging was used to calculate the lung shunt fraction; patients with a lung shunt <10% received the full dose of ^90^Y-microspheres, when lung shunt fraction was between 10%–15% or 15%–20% the dose of ^90^Y-microspheres was reduced with 20% and 40%, respectively. [15] Lung shunt fractions of >20% implied that no treatment could be given. If radioactivity was detected in non-target organs, such as pancreas, duodenum or stomach, further angiographic investigation was performed with additional coiling and/or a more distal injection position of ^99m^Tc-MAA. [16] Patients stayed one night in the hospital for observation.

Treatment angiography was performed within two weeks after the pre-treatment angiography. Patients were readmitted to hospital the day before angiography, where they again received pre- and post-hydration. One hour before angiography, patients received a single intravenous dose of dexamethason (10 mg) and ondansetron (8 mg). The dose of radioactive resin microspheres (SIR-Spheres®, SIRTeX, Lane Cove, Australia) for each individual patient was calculated according to the body surface area method provided by the manufacturer. [15] The tumor volume and total liver volume were calculated by volumetric assessment of CT imaging. Subsequently, the dose of ^90^Y-microspheres was administered with the catheter tip in the hepatic artery or one of its branches, at the same position as used for the injection of ^99m^Tc-MAA. The total liver weight (*m_liver_*) was derived from CT-volumetric measurements assuming a density of 1 kg/l. The net amount of administered radioactivity (*A_net_*) (prepared activity minus residual activity in administration system and catheter) was calculated. The whole liver absorbed dose (*D_liver_*), assuming a homogeneous distribution and full absorption of activity in the liver, was then estimated using the following Medical Internal Radiation Dose (MIRD) committee-based formula [17]:
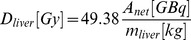



Patients received ^90^Y-RE as a whole liver treatment in a single angiographic procedure (i.e. whole liver delivery), whole liver treatment in two sessions (i.e. sequential delivery) or as treatment of a single lobe (i.e. lobar treatment). In cases of sequential delivery, the aim was to perform both treatment sessions within a commonly accepted interval of 30–45 days. [11] The distribution of ^90^Y-microspheres was assessed with either bremsstrahlung SPECT or ^90^Y-positron emission tomography computed tomography (PET-CT). Our institution’s radiation safety committee required all patients to stay in the hospital for a minimum of 12 hours after treatment.

### Toxicity Assessment

Post-treatment, patients reported to the outpatient clinics at intervals of approximately four weeks. At these visits, physical examination and laboratory tests were performed. The following laboratory investigations were included in our analysis in order to assess laboratory toxicity: total bilirubin, alkaline phosphatase (ALP), gamma-glutamyl transferase (GGT), aspartate aminotransferase (AST), alanine aminotransferase (ALT), albumin, hemoglobin (Hb) and white blood cell count (leukocytes). Blood samples, taken up to four weeks prior to ^90^Y-administration and during a four months follow-up were used for toxicity analysis. Laboratory toxicity was graded according to the Common Terminology Criteria for Adverse Events (CTCAE) v4.0. [18] GGT, AST, ALT and Hb reference values were gender dependent. For each patient, baseline CTCAE grades and maximal CTCAE grades during follow-up were determined. In addition, new toxicity or progression of baseline toxicity to a higher CTCAE grade was grouped separately and will be referred to as “new toxicity“. Patients, in whom data on baseline and/or follow-up laboratory investigations were not available in our center, were excluded from the laboratory toxicity assessment. The clinical toxicity assessment was based on the reporting of periprocedural complications, treatment-related symptoms (CTCAE grade 1–2) and serious adverse events (CTCAE grade 3–4), in the patient’s charts.

### Response Assessment

Baseline imaging was performed with CT or magnetic resonance imaging (MRI) of the liver. In addition, patients with (suspected) ^18^F-fluorodeoxyglucose (^18^F-FDG)-avid tumors received ^18^F-FDG-PET to assess the presence of extrahepatic metastases. Follow-up imaging was performed with CT or MRI of the liver (depending on the modality used for baseline imaging) at approximately 1, 3 and 6 months post-treatment. Response assessment was performed in accordance with the Response Evaluation Criteria in Solid Tumors (RECIST 1.1) on the level of target lesions (TL), whole liver (including non-target lesions) and overall response (including non-target lesions and extrahepatic disease) at 3 months (range 2.0–4.5 months) and 6 months (range 4.5–7.5 months) after the first ^90^Y-RE procedure. [19] Up to five target lesions per patient were identified by an observer (either MS or CR) and the maximal cross-sectional diameter of each target lesion was subsequently measured by the other observer. Observers were blinded for the identity and characteristics of the patient; date of imaging and whether it was a baseline or follow-up scan. Data on progression of non-target lesions, new liver lesions and progression of extrahepatic disease were extracted from radiologic reports. Patients who were lost to follow-up were regarded as having progressive disease (PD) on the ‘overall response level’ at the time of death. Median time to progression (TTP) was calculated for all response levels per Kaplan-Meier analysis.

### Survival Analysis

Overall survival was defined as the interval between the date of (first) ^90^Y-RE treatment and the date of death or most recent contact (alive). Median overall survival (including corresponding 95% CI) was calculated through Kaplan-Meier survival-analysis. Statistical analyses were performed with SPSS Statistics 20.0 for windows (IBM SPSS, Chicago, IL). All percentages were rounded to the nearest whole number.

## Results

### Patients

**Figure 1:**
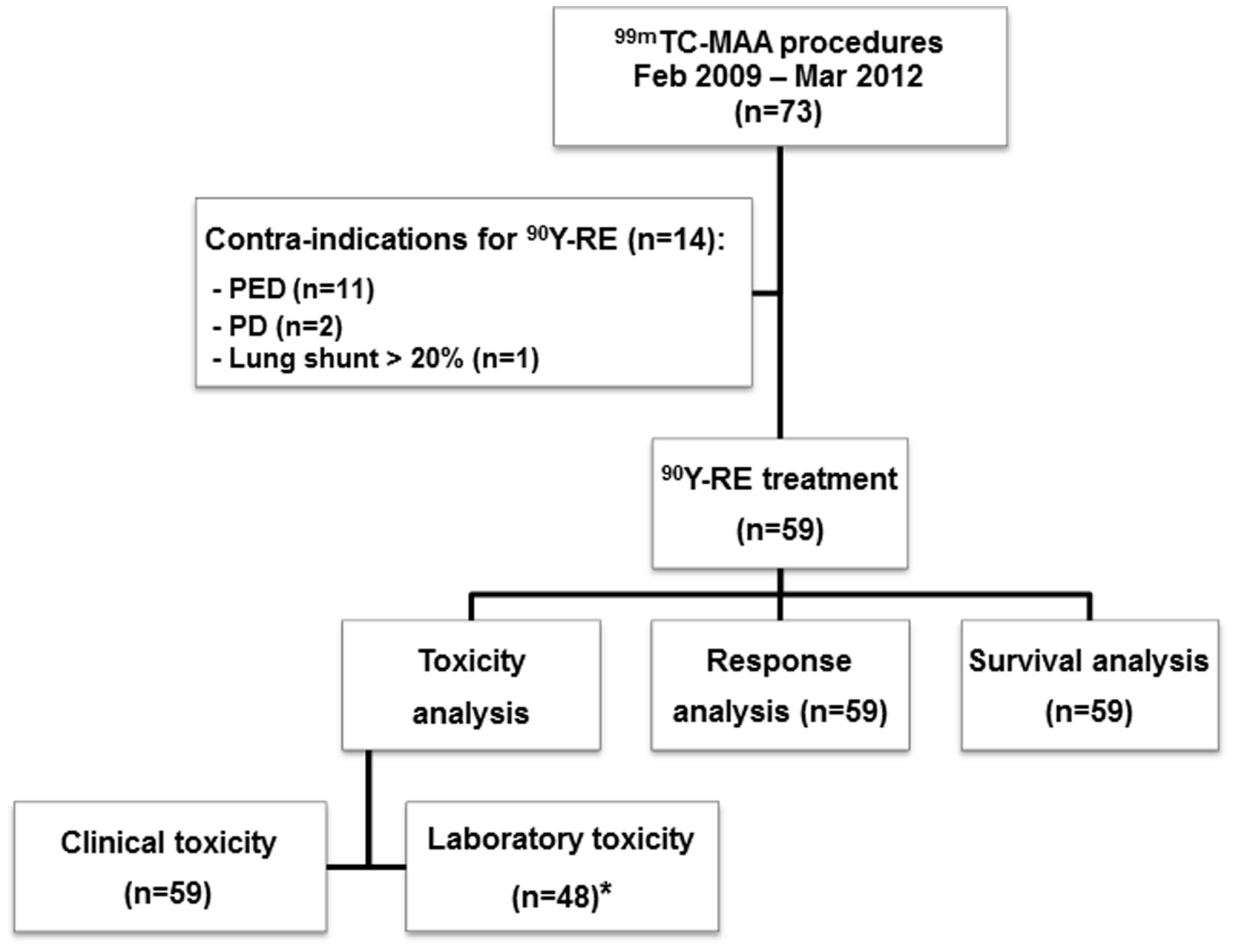
Flowchart. Flowchart displaying treatment selection and study design. *11 patients were non-evaluable for the laboratory toxicity assessment. Abbreviations: PED = persistent extrahepatic deposition; PD = rapidly progressive disease.

Between February 1^st^ 2009 and March 31^st^ 2012, a total of 73 consecutive patients (excluding patients participating in a prospective clinical trial) with liver metastases were considered eligible for ^90^Y-RE treatment at our institute and received a pre-treatment angiographic procedure with ^99m^Tc MAA. A flowchart of the study design and patient treatment is presented in Figure 1. Fourteen patients (19%) could not be treated with ^90^Y-RE, due to persistent extrahepatic deposition (PED) of ^99m^Tc-MAA (n = 11), rapidly progressive disease (n = 2) and a lung shunt fraction exceeding twenty percent (26%, n = 1). Fifty-nine patients received ^90^Y-RE treatment.

Baseline characteristics of these patients are presented in Table 1. The majority of the patients (30/59, 51%) had colorectal cancer liver metastases, six patients (10%) had neuroendocrine tumor (NET) liver metastases, and 23 patients (39%) suffered from liver metastases from various other primary tumors.

**Table 1:** Baseline characteristics.

Baseline Characteristics		Value
Mean age (years)		60±12
Gender		
Male		32 (54%)
Female		27 (46%)
Primary tumor		
Colorectal cancer		30 (51%)
Neuroendocrine cancer		6 (10%)
Uveal melanoma		6 (10%)
Breast cancer		5 (9%)
Esophageal cancer		2 (3%)
Gallbladder cancer		2 (3%)
Gastric cancer		1 (2%)
Pancreatic cancer		1 (2%)
Nasopharyngeal cancer		1 (2%)
Extrahepatic cholangiocarcinoma		1 (2%)
ACUP		2 (3%)
UCC		1 (2%)
GIST		1 (2%)
WHO performance score		
WHO = 0		31 (53%)
WHO = 1		14 (24%)
WHO ≥2		7 (12%)
Unreported		7 (12%)
Child-Pugh score		
A5–A6		53 (90%)
B7–B8		6 (10%)
Tumor burden		
<25%		43 (73%)
≥25%–<50%		11 (19%)
≥50%		5 (9%)
Evidence of extrahepatic metastases		
Yes		16 (27%)
No		43 (73%)
Prior treatment		
Systemic treatment		51 (86%)
Locoregional treatment		50 (85%)
Salvage versus Non-salvage therapy		
Salvage therapy		41 (70%)
Non-salvage therapy		17 (29%)
Unreported		1 (2%)

Values are presented as n (percentage) or mean ± standard deviation. Percentages do not add up to 100%, due to rounding to the nearest whole number. Salvage therapy = 90Y-RE after all regular treatment options have been tried. Non-salvage therapy 90Y-RE, when not all treatment options have been tried yet. Abbreviations: ACUP = Adenocarcinoma of Unknown Primary; UCC = Urothelial Cell Carcinoma; GIST = Gastrointestinal Stromal Tumor; WHO = World Health Organization.

Treatment details are presented in Table 2. The majority of the patients received a whole liver treatment in one session (n = 38, 64%), with a selective administration of ^90^Y-microspheres in the left and right hepatic artery (n = 28) or administration in the proper (n = 9) or common hepatic artery (n = 1). In ten patients, whole liver treatment was performed selectively in sequential sessions (n = 10, 17%), with a median interval of 14 days (range 12–77 days) between both treatment sessions. Eleven patients received unilobar treatment (n = 11, 19%). The mean net administered activity was 1473 MBq (standard deviation 447) with an estimated mean liver-absorbed dose of 42.0 Gy (standard deviation 14.3). Post-treatment bremsstrahlung scintigraphy or ^90^Y-PET, revealed no extrahepatic deposition of radioactivity in any of the patients. Four patients were retreated with ^90^Y-RE after disease progression had occurred, with a median interval of 9 months (range 5–25 months) between the first and second treatment. Median time of hospital admission was 2 days (range 1–4 days). Fifty-four patients (92%) were discharged the day after treatment. The other five patients required longer hospitalization (one or two days extra), due to comorbidities such as renal insufficiency, diabetes mellitus or heart failure.

**Table 2:** Treatment details.

Treatment details	Value
Initial extrahepatic deposition of ^99m^Tc-MAA	12 (20%)
Median ^99m^Tc-MAA lung shunt fraction	6% (0–20%)
Dose reduction required in n patients	3 (5%)
Mean ^90^Y administered activity (MBq)	1473±447
Mean ^90^Y liver absorbed dose (Gy)	42.0±14.3
Whole liver treatment in one session	38 (64%)*
Selective administration (LHA & RHA)	28 (48%)
PHA	9 (15%)
CHA	1 (2%)
Whole liver treatment in sequential sessions(LHA & RHA)	10 (17%)
Lobar treatment	11 (19%)
Retreatment with ^90^Y-RE after progression	4 (7%)
Median time to retreatment (months)	9 (5–25)

Values are presented as n (percentage), median (range) or mean ± standard deviation. * This number includes four patients with a history of previous hemihepatectomy. Abbreviations: MBq = Megabecquerel; Gy = Gray; LHA = Left Hepatic Artery; RHA = Right Hepatic Artery; PHA = Proper Hepatic Artery; CHA = Common Hepatic Artery.

### Toxicity

**Figure 2:**
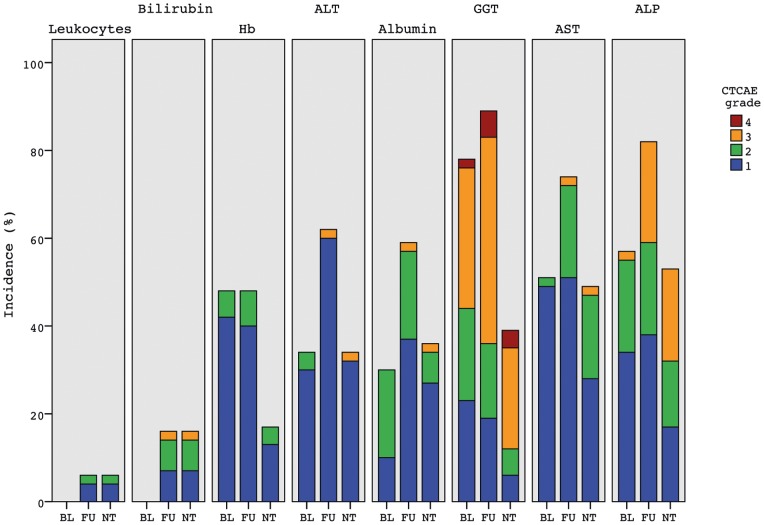
Laboratory toxicity. Clustered bar-chart displaying the incidence of laboratory toxicity at baseline (BL), during follow-up (FU) and corresponding ‘new toxicity’ (NT) per laboratory value. CTCAE grades: blue = grade 1; green = grade 2; orange = grade 3; red = grade 4. Abbreviations: ALT = alanine amino-transferase; Hb = hemoglobin; AST = aspartate aminotransferase; AP = alkaline phosphatase; GGT = gamma-glutamyl transferase.

Eleven patients (19%) were excluded from laboratory toxicity analysis, because data on laboratory investigations at baseline or during follow-up, within our defined intervals, were not available in our center. In the remaining 48 patients, there were values missing for some laboratory parameters, therefore the denominator was adjusted accordingly when calculating incidences. CTCAE grades at baseline, maximum CTCAE grades during follow-up and corresponding new toxicity are presented in Figure 2. Grade 3–4 toxicity at baseline was observed for GGT (16/47, 34%) and ALP (1/47, 1%). Grade 3–4 new toxicity was observed in 18 patients (38%), including following parameters: GGT (13/47, 27%), ALP (10/27, 21%), bilirubin (1/41, 2%), AST (1/47, 2%), ALT (1/47, 2%), and albumin (1/42, 2%). In addition, the incidence of grade 3–4 new toxicity was stratified according to treatment strategy. Ten out of 28 evaluable patients (36%) who received whole liver treatment in one session had grade 3–4 new toxicity, compared to five out of ten patients (50%) who received whole liver treatment in sequential sessions, and three out of ten patients (30%) who received unilobar treatment (Table 3).

**Table 3:** Grade 3/4 laboratory toxicity.

Treatment strategy	Incidence of new grade 3/4 laboratory toxicity
All evaluable patients	18/48 (38%)
Whole liver treatment in one session	10/28* (36%)
Whole liver treatment in sequential sessions	5/10 (50%)
Single lobar treatment	3/10 (30%)

Values are presented as n (percentage).

* This number includes four patients with a history of previous hemihepatectomy.

The following periprocedural complications were reported: allergic reaction to contrast agent (n = 6), arterial dissection (n = 2), nausea/vomitus during angiography (n = 1), delayed hemostasis at the access site requiring prolonged clamping (n = 1), inguinal hematoma at the access site (n = 1). Complications did not prevent any patients from receiving therapy. Back pain or abdominal pain during angiography was managed with fentanyl (37% of patients, range 50–200 mcg i.v.) and/or diclofenac (35% of patients, range 50–125 mg i.v.).

Clinical symptoms associated with the postembolization syndrome (CTCAE grade 1–2) were observed in the majority of the treated patients. This syndrome comprised the following symptoms (in order of frequency): fatigue and loss of appetite, pain/discomfort in the right upper abdominal quadrant requiring analgesics (paracetamol and/or diclofenac and/or morphine), nausea and vomitus, fever and general discomfort. In general, these symptoms started on the day of treatment and lasted up to two weeks after treatment. No grade 3–4 clinical toxicity was observed after^ 90^Y-RE treatment and no serious treatment-related complications such as duodenal or gastric ulceration, radiation pneumonitis or RILD, were observed.

### Response

Target lesions-, whole liver- and overall response rates and TTP (for all patients and per tumor type) at 3- and 6-months are displayed in Table 4. Target lesion, whole liver and overall disease control rates (complete response+partial response+stable disease) at 3-months post-treatment were 35%, 21% and 19% respectively. Corresponding disease control rates at 6-months were 25%, 13% and 12%. Median TTP for all patients was 6.2 months (95% CI 2.2–10.0) for target lesions, 3.3 months (95% CI 2.8–3.8) for the whole liver and 3.0 months (95% CI 2.4–3.5) overall.

**Table 4:** Response Rates and Time to Progression.

	Target Lesion		Whole liver		Overall	
	*3 months*	*6 months*	*3 months*	*6 months*	*3 months*	*6 months*
CR	2 (3%)	0	2 (3%)	0	1 (2%)	0
PR	3 (5%)	3 (5%)	2 (3%)	2 (3%)	2 (3%)	1 (2%)
SD	16 (27%)	12 (20%)	9 (15%)	6 (10%)	8 (14%)	6 (10%)
PD	16 (27%)	6 (10%)	26 (44%)	16 (27%)	30 (51%)	19 (32%)
Deceased	9 (15%)	24 (41%)	9 (15%)	24 (41%)	9 (15%)	24 (41%)
NE	9 (15%)	10 (17%)	7 (12%)	7 (12%)	5 (9%)	5 (9%)
Loss FU	4 (7%)	4 (7%)	4 (7%)	4 (7%)	4 (7%)	4 (7%)
Disease control rate	35%	25%	21%	13%	19%	12%
TTP (all patients)	6.2 months (2.2–10.0)		3.3 months (2.8–3.8)		3.0 months (2.4–3.5)	
TTP (CRLM)	6.2 months (2.5–9.8)		3.0 months (2.8–3.3)		2.8 months (2.2–3.3)	
TTP (NET)	36.4 months (0–88.7)		19.0 months (0–62.0)		11.7 months (0–24.8)	
TTP (Other)	4.4 months (0.8–8.0)		3.8 months (1.9–5.5)		3.3 months (2.2–4.4)	

Values are presented as n (percentage) or median Kaplan-Meier estimate (95% confidence interval). Abbreviations: CR = Complete Response; PR = Partial Response; SD = Stable Disease; PD = Progressive Disease; NE = Non-evaluable; Loss FU = Loss to Follow-Up; TTP = Time To Progression.

### Survival

**Figure 3:**
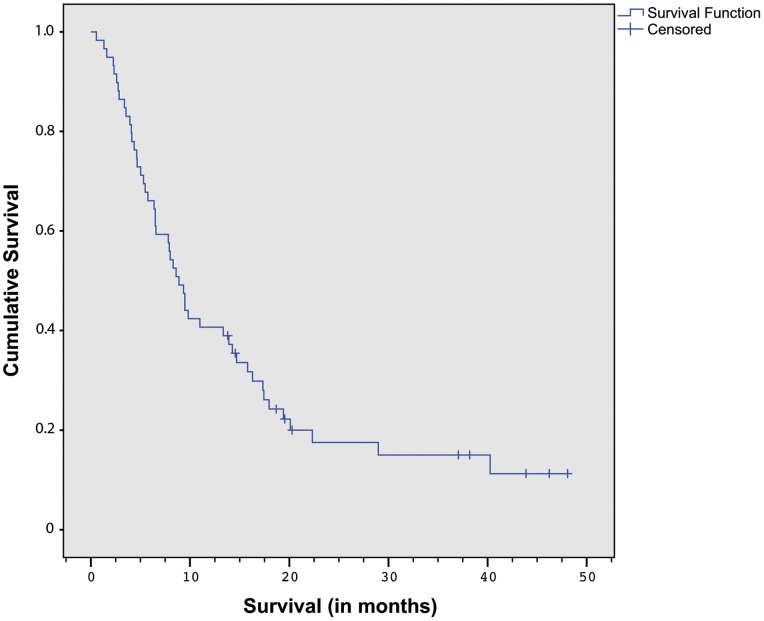
Kaplan-Meier Survival curve for all 59 patients.

**Figure 4:**
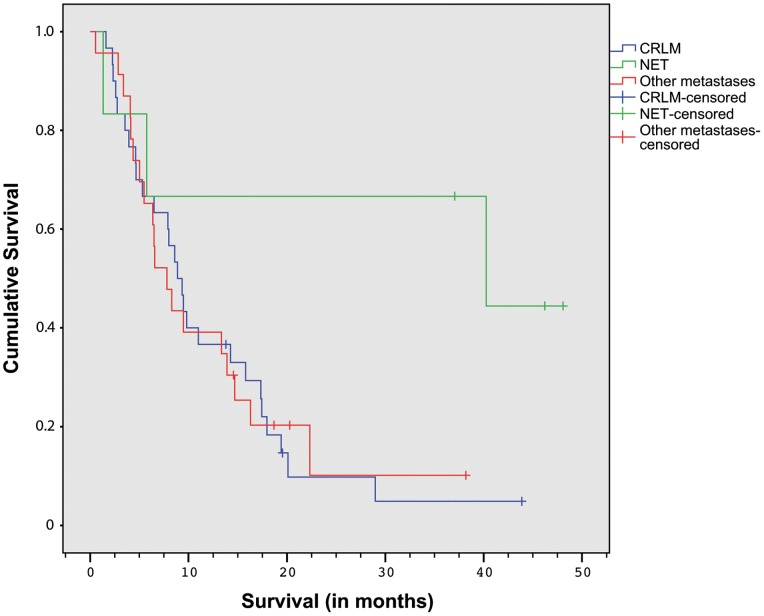
Kaplan-Meier Survival curve per tumor type. The blue line represents patients with colorectal liver metastases (CRLM), the green line represents patients with neuroendocrine tumor (NET) liver metastases, and the red line represents patients with liver metastases from other primary tumors.

At the time of analysis, 49 patients had died and 10 patients were still alive. Median overall survival for the entire group of patients (n = 59) was 8.9 months (95% CI 7.2–10.6). The Kaplan-Meier survival curve is displayed in Figure 3. Median overall survival was 8.9 months (95% CI 6.9–10.9) for colorectal cancer liver metastases (n = 30), 40.3 months (0–107.9) for NET metastases (n = 6) and 7.8 months (95% CI 5.0–10.6) for other metastases (n = 23) (Figure 4).

## Discussion

The primary objective of this study was to investigate treatment-related clinical and laboratory toxicity in patients with unresectable liver metastases, treated with ^90^Y-RE. Secondary objectives were to assess tumor response and overall survival. Clinical toxicity was confined to grade 1–2 symptoms of the post-embolization syndrome. No RILD or other grade 3–4 clinical toxicity was observed, whereas laboratory toxicity grade 3–4 was observed in 38% of patients. In this cohort, a disease control rate of up to 35% was obtained at 3-months post-treatment, and median overall survival was 8.9 months.

Tumor response rates vary widely in the ^90^Y-RE literature. [4] This may be explained in part by differences in methodology for response assessment. Various studies do not specify whether RECIST criteria have been followed. According to these criteria, tumor response should be differentiated in target lesion, liver and overall response. [19] In order to improve interpretability of overall response rates, studies should indicate whether patients had evidence of extrahepatic disease at baseline. Response rates are commonly divided into 3- and 6-months rates post-treatment. However, it should be clearly stated which imaging intervals are chosen to represent this 3- and 6-months measurements. In addition, it would be preferable to score target lesion response blindly, to assure objective measurements. In a comprehensive review of the ^90^Y-RE literature, twelve studies were identified that reported a 3-month disease control rate, ranging from 63–100%. [4] In most of these studies, the level on which response assessment had been performed was not specified. Assuming these are whole-liver disease control rates, our 3-month disease control rate was much lower: 21%. This difference could be attributable to differences in methodology of response assessment, as mentioned above. However, less stringent patient selection criteria and the heterogeneity of our cohort, including hyper- and hypovascular liver metastases from various primary tumors, could also have attributed to lower response rates.

Toxicity due to radiation to the liver has first been described after external radiation therapy. [20], [21] It was found that the liver is very sensitive to radiation and patients may develop radiation induced liver disease (RILD), months after an overdose of radiation. Histopathologically, RILD is characterized by veno-occlusive disease with congestion of the central veins and sinusoids.[21]–[24] The symptoms of RILD comprise fatigue, anicteric ascites, hepatomegaly, and elevated liver function tests (especially alkaline phosphatase). [23] High dose corticosteroids can be given to mitigate the course of this disease. It is however, hard to recognize RILD since it has a long latency time and many of its symptoms can also occur after non-complicated treatment with ^90^Y-RE. A better understanding of the physiological variation of treatment-related laboratory toxicity after ^90^Y-RE would be very helpful in discriminating early signs of RILD from transient laboratory abnormalities after treatment. Mild toxicity (grade 1–2) of liver function tests is common after ^90^Y-RE, occurring in up to 70% of the patients.[25]–[27] Reported incidences of grade 3–4 toxicity are much lower and vary widely across studies. Van Hazel *et al.* [2] observed no grade 3–4 toxicity in their study, Piana *et al.* [25] found an overall incidence of 7% and Kennedy *et al.* [8] reported an incidence of up to 20.5% for ALP. In the study of Piana *et al.*, one patient died of RILD. [25] In our study we found higher incidences of laboratory toxicity, with new laboratory toxicity grade 3–4 occurring in up to 38% of the patients. However, we did not observe any serious treatment-related complications, nor did we observe any RILD. This indicates that serious laboratory toxicity regarding transaminases and liver function tests can occur as part of the physiological reaction of the liver to ^90^Y-RE treatment.

One of the factors complicating the interpretation of toxicity results is that abnormalities in liver function tests and transaminases could be the result of tumor progression instead of treatment-related toxicity. Moreover, results of toxicity are often incompletely reported in the ^90^Y-RE literature. Many studies do not specify how CTCAE scores for laboratory toxicity have been determined. This could inadvertently lead to an underestimation of treatment toxicity and it limits the comparability of studies. Therefore, we aimed to report our methods and results in an unambiguous and transparent fashion.

The most important limitations of this study were its retrospective design and the lack of standardization of laboratory investigations and reporting of clinical symptoms during physical examination. Therefore, our results in terms of the incidence of laboratory or clinical toxicity are likely to be underestimations of the real incidence of toxicity. Another limitation was the heterogeneity of our study population. However, this heterogenic group does reflect the typical population of patients referred for ^90^Y-RE treatment.

Fourteen of the 73 patients (19%) who received work-up angiography did not receive ^90^Y-RE. The majority of these patients (n = 11) were not eligible because of persisting extrahepatic deposition (PED) of ^99m^Tc-MAA. This PED rate of 11/73 (15%) is much higher than the rates reported in the literature (ranging from 0% to 10%). [16], [28], [29] A likely cause of the high PED rate in this study is the relative large number of proximal injection positions (i.e. proper or common hepatic artery). Several studies have demonstrated that extrahepatic deposition can be solved/prevented by more distal injection positions (left/middle/right hepatic artery or even more selective). [16], [28], [30] We have changed our current practice accordingly and we rarely perform whole liver treatments from the proper hepatic artery anymore. In addition, our center and many others increasingly use c-arm cone beam computed tomography during the pre-treatment angiography to help prevent extrahepatic distribution and identify culprit vessels. [31], [32].

The whole liver approach has also been associated with increased toxicity. Seidensticker *et al.* have reported that a whole liver approach, in non-cirrhotic liver metastases patients, resulted in a higher number of liver-related CTCAE grade 3–4 events as compared to a sequential lobar approach. [14] We could not confirm this finding in our patients. In fact, the number of patients with CTCAE grade 3–4 laboratory toxicity was even lower in the whole liver approach group (36%) than in the sequential lobar group (50%). Selection bias, and confounding due to differences in baseline characteristics, may play a significant role in this matter. However, we do recognize the clinical importance, and we think that the question whether treating the whole liver at once increases toxicity, should be determined using a randomized controlled trial.

The majority of the patients (70%) treated in our cohort, received radio-embolization as salvage therapy. This illustrates that ^90^Y-RE is still regarded as a treatment option of last resort, for patients who have unresectable and chemorefractory liver tumors. The costs of radioembolization treatment (approximately **€**11.000 for one dose of SIR-spheres plus the costs of the procedure, the involved imaging, hospitalization and follow-up) need to be weighed against the potential benefit to the patient. [33] For this purpose, prospective comparative studies evaluating survival, tumor response, and quality of life after ^90^Y-RE are strongly warranted. In addition, it will become increasingly important to select those patients that will benefit most from this therapy. Performing radioembolization at an earlier stage in patients with liver metastases might for instance translate into improved tumor response rates and overall survival. Two large randomized controlled trials are currently ongoing, investigating the effect on overall survival (SIRFLOX study) and progression free survival (FOXFIRE study) of the addition of ^90^Y-RE to FOLFOX (fluorouracil, leucovorin, oxaliplatin) with or without bevacizumab as first-line treatment for patients with unresectable colorectal liver metastases. [34].

### Conclusion

The risk of severe complications or grade 3–4 clinical toxicity in patients with liver metastases of various primary tumors undergoing ^90^Y-RE is low. In contrast, laboratory toxicity grade 3–4 was observed in more than one-third of the patients without any signs of RILD. This physiological reaction of liver enzymes to ^90^Y-RE therapy may mask early signs of toxicity due to RILD.
