# Correction: Clinical and Laboratory Toxicity after Intra-Arterial Radioembolization with ^90^Y-Microspheres for Unresectable Liver Metastases

**DOI:** 10.1371/annotation/559e04cc-b09c-4b5c-9405-56837f6d5627

**Published:** 2013-10-23

**Authors:** Maarten L. J. Smits, Andor F. van den Hoven, Charlotte E. N. M. Rosenbaum, Bernard A. Zonnenberg, Marnix G. E. H. Lam, Johannes F. W. Nijsen, Miriam Koopman, Maurice A. A. J. van den Bosch

All authors except for the seventh author are affiliated only with institution number 1, Department of Radiology and Nuclear Medicine, University Medical Center Utrecht, Utrecht, The Netherlands; they are not additionally affiliated with institution number 2. 

